# Associations between Zygoma Fracture and Post-Traumatic Headache: A Study among Taiwanese Population

**DOI:** 10.3390/jcm10225377

**Published:** 2021-11-18

**Authors:** I-Han Hsiao, Shao-Yun Hsu, Mei-Chen Lin, Pin-Keng Shih

**Affiliations:** 1 Department of Neurosurgery, China Medical University Hospital, Taichung 404, Taiwan; coolfishing2002@gmail.com; 2 Graduate Institute of Acupuncture Science, China Medical University, Taichung 404, Taiwan; 3 Division of Reconstructive Microsurgery, Department of Plastic and Reconstructive Surgery, Chang Gung Memorial Hospital, College of Medicine, Chang Gung University, Taoyuan 333, Taiwan; fishy9681@gmail.com; 4 School of Medicine, China Medical University, Taichung 404, Taiwan; coolindm@gmail.com; 5 Management Office for Health Data, China Medical University and Hospital, Taichung 404, Taiwan; 6 Department of Surgery, China Medical University Hospital, Taichung 404, Taiwan

**Keywords:** post-traumatic headache, zygoma fracture

## Abstract

Few studies have discussed the development of post-traumatic headache (PTH) after zygoma fracture. This research aimed to examine the association between zygoma fracture and PTH and its other associated factors. A total of 3043 patients with zygoma fracture and 3043 patients with non-fracture were included in this analysis. They were matched to a non-fracture cohort from the National Health Insurance database according to age, sex, and index year. The incidence of PTH and its association with zygoma fracture were assessed. The zygoma fracture cohort had a significantly higher cumulative incidence of PTH than the non-fracture cohort in a 10-year follow-up. The confounding risk factors of PTH included zygoma fracture, female sex, and comorbidities, including obesity and depression. Female patients under 40 years old who had zygoma fractures had a higher incidence of PTH than the non-fracture group. Moreover, patients with zygoma fractures commonly developed PTH within three months after injury. Female patients under 40 years old with precedent zygoma fractures had a higher incidence rate of PTH than those without fractures. Moreover, patients with zygoma fractures commonly developed PTH within three months after injury. Nevertheless, before widely applying our results, a prospective study must be conducted to verify the risk factors found in this study.

## 1. Introduction

Post-traumatic headache (PTH) is defined as headache developing within seven days after precedent trauma or regaining consciousness from traumatic consciousness loss [1]. Moreover, it is commonly caused by traumatic brain injury (TBI) and is characterized by migraine or tension-type headaches [2]. The incidences of PTH in patients with moderate/severe and mild TBI are 71% and 54%, respectively [3]. The current treatment focuses on physical therapy, neurostimulation, Botox (onabotulinum toxin A) injection, and surgery [4].

In addition to PTH, trauma is also the predominant precipitating factor of post-traumatic migraine (PTM), and the condition is characterized by headache, nausea, and either photophobia (sensitivity to light) or phonophobia (sensitivity to noise) [5]. Weiss, H.D. et al. showed the occurrence of subsequent PTM after minor head or neck injuries in patients without a previous history of headache [6]. Mihalik, J.P. et al. revealed that the PTM group had more significant neurocognitive deficits than the headache and non-headache groups [7]. Even though the underlying mechanisms remain unclear, PTM is highly suspected to be attributed to changes in neuronal depolarization, excitatory amino acid levels, inhibitory neurotransmitters, and other biochemical mechanisms [8].

A previous study showed that malar and nasal fractures were significantly correlated with subsequent migraine, and there were no significant differences in the incidence of migraine among patients who underwent surgeries [9]. Despite this, forward-looking zygoma fracture and PTH analysis are not commonly performed, and the associated risk factors are not well studied. Hence, the current study aimed to investigate the association between PTH and zygoma fracture.

## 2. Materials and Methods

### 2.1. Data Source

The National Health Insurance Program has enrolled nearly 99% of the Taiwan population and documented their medical records in the National Health Insurance Research Database (NHIRD) since 1995. The database includes all medical records comprising visits to clinics, emergency departments, hospitalizations, operations, and each medical service of every medical institution in Taiwan. Admission-based files were used to conduct this study. All individuals with at least one hospitalization for corresponding diagnosis identification numbers (International Classification of Diseases, Ninth Revision, Clinical Modification, ICD-9-CM: 802.4 and 802.5) were included. The diagnosis identification numbers are defined based on the International Classification of Disease, Ninth Revision, Clinical Modification (ICD-9-CM). The Research Ethics Committee of China Medical University and Hospital in Taiwan approved this study (CMUH-104-REC2–115-(CR6)).

### 2.2. Study Population

We conducted a retrospective cohort study to assess the association between zygoma fracture and PTH. Patients hospitalized due to zygoma fracture (ICD-9-CM: 802.4 and 802.5) from 2000 to 2012 were enrolled in this study and included in the exposed group. In addition, patients without any trauma history based on the hospital records were classified into the unexposed group.

The index date was defined as when the patient was initially diagnosed with zygoma fracture or the reasonable date during the insurance period in the exposed and unexposed groups, respectively. The primary outcomes of this study were newly occurred migraines (ICD-9-CM: 346) and/or tension headaches (ICD-9-CM: 307.81) that required hospital admissions. The study period started from the index date to the date of initial diagnosis of migraine, withdrawal from NHIRD, or to the endpoint of our approval, December 31, 2013. Patients diagnosed with migraine and tension headache before the index date or under 18 years old (calculated until the index date) were excluded from this study.

To minimize the influences of confounding factors, we paired each patient with zygoma fracture with one patient in the unexposed group with propensity score matching. The confounding factors include age, sex, index year, and comorbidities such as hypertension (codes 401–405) [10], diabetes (code 250) [11], hyperlipidemia (ICD-9-CM: 272) [12], subdural hemorrhage (ICD-9-CM: 432.1) [13], obesity (ICD-9-CM: 278) [14], alcoholism (ICD-9-CM: 291, 303, 305, and V113) [15], and depression (ICD-9-CM: 296.2, 296.3, 296.82, 300.4, 309.0, 309.1, 309.28, and 311) [11].

### 2.3. Diagnosis of PTH

Based on the diagnostic criteria of PTH by the International Classification of Headache Disorders-3 (ICHD III) [1], post-traumatic headache, and head and/or neck injuries are defined as headaches that developed within seven days after the precedent trauma, regaining consciousness from the post-traumatic immediate consciousness loss, and recovered alertness and expression of pain. Because the International Classification of Diseases did not have a specific code for PTH since the beginning of the databank, the codes for migraine (ICD-9-CM: 346) and tension headache (ICD-9-CM: 307.81) are applied in this study as the diagnosis code for PTH.

### 2.4. Statistical Analysis

The distribution of demographic characteristics and comorbidities between the two cohorts was presented. To compare the difference between the two cohorts, we used the *t*-test and the chi-square test to assess continuous and categorical variables, respectively.

To validate the association between zygoma fracture and the risk of PTH, we conducted a Cox proportional hazard regression analysis. Data were presented as hazard ratios (HRs), adjusted hazard ratios (aHRs), and 95% confidence intervals (CIs). The Kaplan–Meier method was used to show the cumulative incidence curves of PTH between the two cohorts, and the difference between the two curves was assessed using the log-rank test. All statistical analyses were performed using SAS version 9.4 (SAS Institute Inc., Cary, NC, USA) and were plotted using R. A two-sided *p*-value of 0.05 was considered statistically significant.

## 3. Results

This study enrolled 6086 participants, and they were classified into the exposed and unexposed cohorts. Approximately 69% of participants were men, and the dominant age group was < 40 years. The mean age of the participants was 40.4 years. Moreover, there were no significant differences in age, sex, or hospital-treated comorbidities after propensity score matching (Table 1).

Table 2 shows the association between the possible risk factors and the risk of PTH. Patients with zygoma fracture (aHR = 1.36, 95% CI: 1.11–1.66), male sex (aHR = 0.54, 95% CI: 0.44–0.67), obesity (aHR = 2.83, 95% CI: 1.16–6.93), and depression (aHR = 2.21, 95% CI: 1.55–3.16) had a significantly higher risk of PTH.

To validate the association between zygoma fracture and a higher risk of PTH in the different subgroups, we conducted a multivariate stratification analysis (Table 3). Female patients with zygoma fracture who were aged < 40 years had a significantly higher risk of PTH (aHR = 1.52, 95% CI: 1.01–2.29).

Moreover, we assessed the association between zygoma fracture and a higher risk of PTH according to the duration of follow-up periods (Table 4). Patients with zygoma fracture who were followed up for < 3 months had a higher risk of PTH (aHR = 2.65, 95% CI: 1.18–5.99).

**Figure 1:**
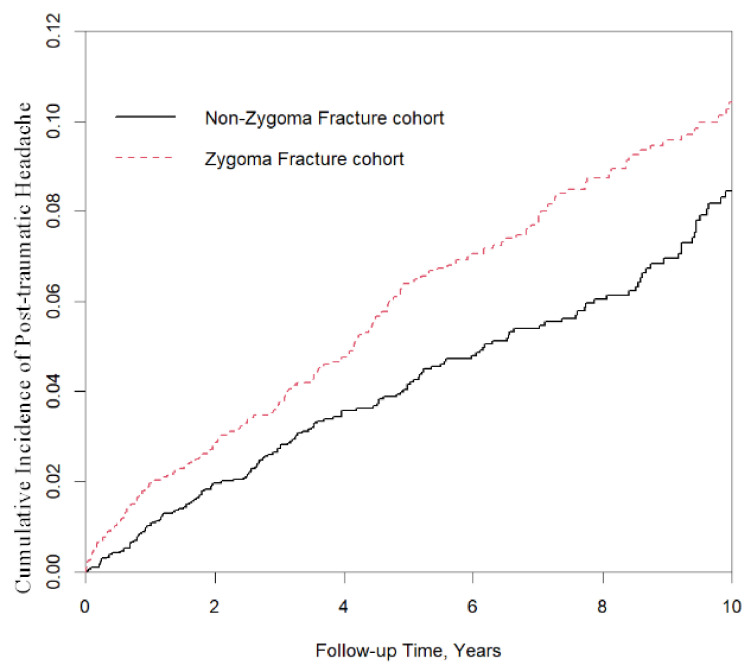
The zygoma fracture cohort had a significantly higher cumulative incidence of post-traumatic headache than the non-fracture group (log-rank test; *p* = 0.004).

Figure 1 shows the cumulative incidence curves of PTH in the zygoma fracture and non-zygoma fracture cohorts. Results showed that patients with zygoma fracture had a significantly higher cumulative incidence of PTH (*p*-value = 0.004).

## 4. Discussion

Based on our results, the risk factors of PTH are zygoma fracture, female sex, and comorbidities including obesity and depression (Table 2). After stratification with various variables, female patients with a fracture under 40 years old had a higher incidence of PTH (Table 3). In addition, PTH commonly developed within three months after injury (Table 4).

Mihalik, J.P. et al. and Weiss, H.D. indicate that PTH may be associated with head/neck injury [6], traumatic brain injury [5], or brain contusion [7]. To decrease bias or the overestimation of incidence rates in this survey, physicians coded all participants only for zygoma fracture if there was no evidence to prove that they had other injuries.

The outcomes in this study suggested zygoma fracture, female sex, obesity, and depression were significant confounding factors (Table 2). Jeyagurunathan, A. et al. reported the risk factors of migraine, including female sex, young age (<34 years old), and psychiatric conditions (major depressive, bipolar, generalized anxiety, and alcohol abuse disorders) [16]. The similar risk factors found in Jeyagurunathan’s and our study reveal some of the possible crossed pathways and mechanisms between PTH and migraine-type headaches. Nevertheless, our results also showed some risk factors which have not been reported. Zygomatic-related PTH may have more variable inducing mechanisms than the migraine-type headache.

A new hypothesis showed that zygoma-correlated PTH might be associated with auriculotemporal nerve (ATN) and zygomaticotemporal nerve (ZTN) injury [17,18]. Anatomically, both nerve pathways are extremely close to the zygomatic arch. Hence, zygoma fracture-induced PTH may be caused by injury-induced temporal swelling, which can compress the distal route of the ATN and ZTN [19]. In addition, surgical scars resulting from craniotomy or plastic surgeries were the most common damaging mechanism [20]. At present, nerve decompression surgery [21], peripheral nerve blockade [22], and botulinum toxin injection [23] are widely used for managing distal nerve compression pressure.

Another hypothesis is that PTH may result from abnormal signaling in any part of the trigeminal nerve system. A similar study suggests that minor trauma of the head, neck, and craniocervical junction could have a major impact on the vestibular system at variable sites, [24] which may explain the abnormal signals transmitting from a diseased labyrinth to the healthy, normally functioning vestibular nuclei complex in multiple chronic canal benign paroxysmal positional vertigo [25]. Because both ATN and ZTN are proximal to the vestibular nuclei complex, the mechanisms may be the closest analogy to PTH.

Female patients younger than 40 years old with zygomatic fractures have a higher incidence of PTH (Table 3). Peterlin, B.L. et al. suggest that PTH incidence is up to three times higher in females than males because of the effect of gonadal hormones [26]. In brief, we may assume that menstrual hormones enhanced the impact of zygoma fracture on PTH. Hormonal changes associated with puberty and the menstrual cycle may significantly influence migraine-type headaches in young women, and oral contraceptives and pregnancy had effects on the migraine-type headache to some degree [27,28]. Furthermore, retrospectively, migraine was 1.7 and 2.5 times more likely to occur during the two days before and the first three days of menstruation [29]. For some PTH patients comorbid with migraine-type headaches, hormone therapy might be an option for young female patients with zygoma-fracture-related PTH.

Based on the stratification analysis according to different follow-up periods, the incidence of PTH within three months after zygoma fracture was 2.65-fold (Table 4). The lack of significant differences in terms of follow-up periods might indicate that PTH could progressively improve. Hence, zygoma fracture-induced PTH is temporary. In addition, there may be a concern that the high incidence of PTH three months after trauma did not match the definition of PTH, which may be attributed to two reasons. On the one hand, the mixed diagnosis did not reflect the true condition of PTH diagnosis. The PTH diagnosis (ICD-9-CM: 339.2) was not established until 2009. Second, the patients may have had early onset of symptoms but delayed admission for treatment [30] (the medical record in this databank came from the admission data), which implies the timing of medical record was different from that of symptoms onset. The timing discrepancy may lead to misinterpretation of the findings, and readers should be alert to this bias.

To the best of our knowledge, this is the first large-scale and long-term follow-up study to investigate the relationship between zygoma fracture and PTH. At the end of the 10-year follow-up period, the cumulative incidence of PTH was significantly higher in the zygoma fracture cohort than in the matched cohort (Figure 1). Nevertheless, a prospective study of indications based on these findings should be further performed.

The current study had several limitations. Firstly, data about lifestyle habits such as smoking, diet, socioeconomic status, injury mechanisms, drug use, and previous trauma history were not obtained to adjust the risk of developing PTH. Secondly, the associated clinical intervention data, such as operative imaging, laboratory results, and severity/grade of PTH or fracture were not recorded because all data used were anonymized. Third, the prevalence of PTH and fracture might be underdiagnosed and underestimated because all patients were enrolled based on admission data. Fourth, the mixed diagnosis of migraine (ICD-9-CM: 346) and tension headache (ICD-9-CM: 307.81) was used in replace of the code for PTH (ICD-9-CM: 339.2), which may be not reflective of the true condition and may be easily misinterpreted. The medical records in this databank commenced in 2001, but the diagnosis code was not established until 2009. Fifth, biases caused by the retrospective nature of this study such as the timing discrepancy between symptoms onset and medical record should also be considered.

In summary, female patients with zygoma fracture who are younger than 40 years old have a higher incidence rate of PTH than those without fracture. Further, PTH is commonly developed within three months after injury.

## Figures and Tables

**Table 1:** Demographic characteristics and comorbidities of patients newly diagnosed zygoma fracture in Taiwan during 2000–2012.

Characteristics	Total	Zygoma Fracture		*p*-Value
		No *n* = 3043	Yes *n* = 3043	
Gender				0.91
Female	1882	939 (30.9)	943 (31)	
Male	4204	2104 (69.1)	2100 (69)	
Age				0.99
<40	3272	1634 (53.7)	1638 (53.8)	
40–64	2035	1018 (33.5)	1017 (33.4)	
≥65	779	391 (12.8)	388 (12.8)	
Mean (SD) ^a^		40.4 (18.9)	40.4 (18.8)	0.96
Baseline comorbidity				
Hypertension	1127	561 (18.4)	566 (18.6)	0.87
Diabetes mellitus	633	313 (10.3)	320 (10.5)	0.77
Hyperlipidemia	828	418 (13.7)	410 (13.5)	0.76
Gout	9	3 (0.1)	6 (0.2)	0.32
Subdural hemorrhage	566	285 (9.4)	281 (9.2)	0.86
Obesity	35	18 (0.6)	17 (0.6)	0.87
Alcoholism	233	116 (3.8)	117 (3.8)	0.95
Depression	375	188 (6.2)	187 (6.1)	0.96

Chi-square test, ^a^ *t*-test.

**Table 2:** Cox model measured hazard ratio and 95% confidence intervals of post-traumatic headache associated with and without zygoma fracture.

Characteristics	Event	Crude		Adjusted	
	(*n* = 378)	HR (95% CI)	*p* Value	HR (95% CI)	*p* Value
Zygoma Fracture					
No	165	Ref.		Ref.	
Yes	213	1.35 (1.10–1.65)	0.004	1.36 (1.11–1.66)	0.003
Gender					
Female	176	Ref.		Ref.	
Male	202	0.54 (0.44–0.66)	<0.001	0.54 (0.44–0.67)	<0.001
Age at baseline					
<40	228	Ref.		Ref.	
40–64	115	0.89 (0.71–1.11)	0.302	0.78 (0.61–1.00)	0.050
≥65	35	0.89 (0.62–1.27)	0.519	0.68 (0.44–1.05)	0.085
Baseline comorbidity					
Hypertension	64	1.11 (0.85–1.46)	0.436	1.26 (0.89–1.80)	0.193
Diabetes mellitus	28	0.85 (0.58–1.24)	0.391	0.67 (0.43–1.05)	0.082
Hyperlipidemia	49	1.14 (0.84–1.54)	0.398	1.08 (0.72–1.62)	0.717
Gout	30	0.98 (0.68–1.43)	0.927	1.20 (0.83–1.75)	0.329
Obesity	5	3.09 (1.28–7.47)	0.012	2.83 (1.16–6.93)	0.023
Alcoholism	11	0.93 (0.51–1.70)	0.824	0.84 (0.45–1.57)	0.590
Depression	37	2.16 (1.53–3.03)	<0.001	2.21 (1.55–3.16)	<0.001

Abbreviation: HR, hazard ratio; CI, confidence interval. Adjusted HR: adjusted for gender, age, and comorbidities in Cox proportional hazards regression.

**Table 3:** Incidence rates, hazard ratio and confidence intervals of post-traumatic headache in different stratification.

Variables	Matched Cohort			Zygoma Fracture			HR			
	*n* = 3043			*n* = 3043			Crude	*p*-Value	Adjusted	*p*-Value
	Event	Person Years	IR	Event	Person Years	IR	(95% CI)		(95% CI)	
Gender										
Female	80	6192	129.20	96	5941	161.58	1.25 (0.93–1.68)	0.147	1.26 (0.94–1.70)	0.126
Male	85	13,212	64.34	117	12,599	92.86	1.44 (0.99–1.91)	0.010	1.44 (0.99–1.90)	0.111
Age at baseline										
<40	91	11,184	81.36	137	10,794	126.92	1.56 (1.20–2.03)	0.001	1.57 (1.20–2.05)	0.001
Female	39	3157	123.52	56	3032	184.72	1.49 (0.99–2.25)	0.055	1.52 (1.01–2.29)	0.047
Male	52	8027	64.78	81	7763	104.35	1.61 (0.93–2.28)	0.007	1.62 (0.94–2.29)	0.107
40–64	63	6287	100.20	52	6046	86.01	0.86 (0.59–1.24)	0.411	0.86 (0.60–1.24)	0.423
≥65	11	1932	56.93	24	1700	141.16	2.41 (0.88–4.92)	0.016	2.44 (0.89–4.99)	0.315
Baseline comorbidity										
Hypertension	29	2984	97.17	35	2767	126.50	1.29 (0.79–2.11)	0.312	1.30 (0.79–2.13)	0.299
Diabetes mellitus	11	1648	66.74	17	1563	108.79	1.63 (0.77–3.49)	0.205	1.63 (0.76–3.50)	0.211
Hyperlipidemia	22	2205	99.75	27	2101	128.50	1.27 (0.72–2.23)	0.405	1.08 (0.53–2.20)	0.843
Gout	15	1547	96.99	15	1464	102.48	1.05 (0.52–2.16)	0.887	1.28 (0.73–2.26)	0.385
Obesity	2	97	206.71	3	62	481.06	1.83 (0.30–10.96)	0.511	2.04 (0.31–13.44)	0.458
Alcoholism	5	616	81.23	6	530	113.23	1.37 (0.42–4.48)	0.607	1.43 (0.43–4.76)	0.559
Depression	16	934	171.31	21	830	253.09	1.50 (0.78–2.87)	0.226	1.47 (0.76–2.82)	0.252

Abbreviation: IR, incidence rates, per 10,000 person-years; HR, hazard ratio; CI, confidence interval. Adjusted HR: adjusted for gender, age, and comorbidities in Cox proportional hazards regression.

**Table 4:** Incidence rates, hazard ratio and confidence intervals of post-traumatic headache in different follow-up stratification.

Variables	Matched Cohort			Zygoma Fracture			Compared to Non-Zygoma Fracture			
	*n* = 3043			*n* = 3043			Crude HR	*p*-Value	Adjusted HR	*p*-Value
	Event	Person years	IR	Event	Person years	IR	(95% CI)		(95% CI)	
Follow-up time										
<3 month (s)	8	760	105.33	21	749	280.21	2.66 (1.18–6.00)	0.019	2.65 (1.18–5.99)	0.019
3–6 months	5	756	66.16	12	742	161.82	2.44 (0.86–6.94)	0.093	2.45 (0.86–6.96)	0.087
6 months–1 year	18	1501	119.89	26	1464	177.60	1.48 (0.81–2.70)	0.200	1.50 (0.82–2.74)	0.187
≥1 year	134	16387	81.77	154	15,586	98.81	1.21 (0.96–1.52)	0.109	1.22 (0.97–1.54)	0.096

Abbreviation: IR, incidence rates, per 10,000 person-years; HR, hazard ratio; CI, confidence interval. Adjusted HR: adjusted for gender, age and comorbidities in Cox proportional hazards regression.

## Data Availability

This study used inpatient claims data from the Taiwan National Health Insurance Research Database (NHIRD). This database contains detailed medical histories of the hospitalized enrollees in Taiwan. Based on the guideline of Taiwan Ministry of Health and Welfare (TMHW), only citizens of the Taiwan are eligible to apply the NHIRD for research projects (https://nhird.nhri.org.tw/en/Data_Protection.html, accessed on 17 September 2019). The database we applied is limited to our research purpose. All applicants must follow the Computer-Processed Personal Data Protection Law and related regulations of National Health Insurance Administration and NHRI (http://www.winklerpartners.com/?p=987, accessed on 17 September 2019). The NHIRD is owned by TMHW, and the right to use belongs to the researchers. However, other researchers are able to request data access following the regulations of TMHW.
